# Commentary: Long-term in vivo microscopy of CAR T cell dynamics during eradication of CNS lymphoma in mice

**DOI:** 10.3389/fimmu.2020.01503

**Published:** 2020-07-21

**Authors:** Hocine Rachid Hocine, Hue T. Quach, Prasad S. Adusumilli

**Affiliations:** ^1^Thoracic Service, Department of Surgery, Memorial Sloan Kettering Cancer Center, New York, NY, United States; ^2^Center for Cell Engineering, Memorial Sloan Kettering Cancer Center, New York, NY, United States

**Keywords:** solid tumors, blood-brain barrier, CAR T cells, regional administration, CNS lymphoma

## Introduction

Diffuse large B-cell lymphoma (DLBCL), an aggressive extranodal non-Hodgkin lymphoma, accounts for 90% of cases of primary central nervous system lymphoma (PCNSL). Despite treatment with chemotherapy and immunotherapy, 1 in 3 patients with DLBCL either have refractory disease or experience relapse (1, 2). Anti-CD19 CAR T cells have elicited dramatic responses in hematological malignancies, but their efficacy against solid tumors and hematological malignancies with intracerebral tumors remains limited. The hurdles to achieving antitumor efficacy against solid tumors using CAR T cells—such as limited infiltration, limited penetration, and an immunosuppressive microenvironment—are also impediments to eradicating intracerebral tumors in PCNSL (3). In addition, the blood-brain barrier impedes intravenous CAR T-cell administration.

## Novelty

The authors developed a mouse model of orthotopic, fluorescent-labeled PCNSL with intracerebral and periventricular tumor growth and created chronic cranial windows, which allowed longitudinal observation of tumor and T-cell kinetics (4). Three-dimensional quantification of intratumoral T cells by use of two-photon laser scanning microscopy (TPLSM) demonstrated that intravenously administered anti-CD19 CAR T cells accumulated in limited numbers at a late time point, at levels equal to mock CAR T cells, resulting in tumor regression in a fraction of treated mice. In contrast, intracerebrally administered anti-CD19 CAR T cells accumulated and penetrated deeply at higher numbers than mock CAR T cells.

**Figure 1:**
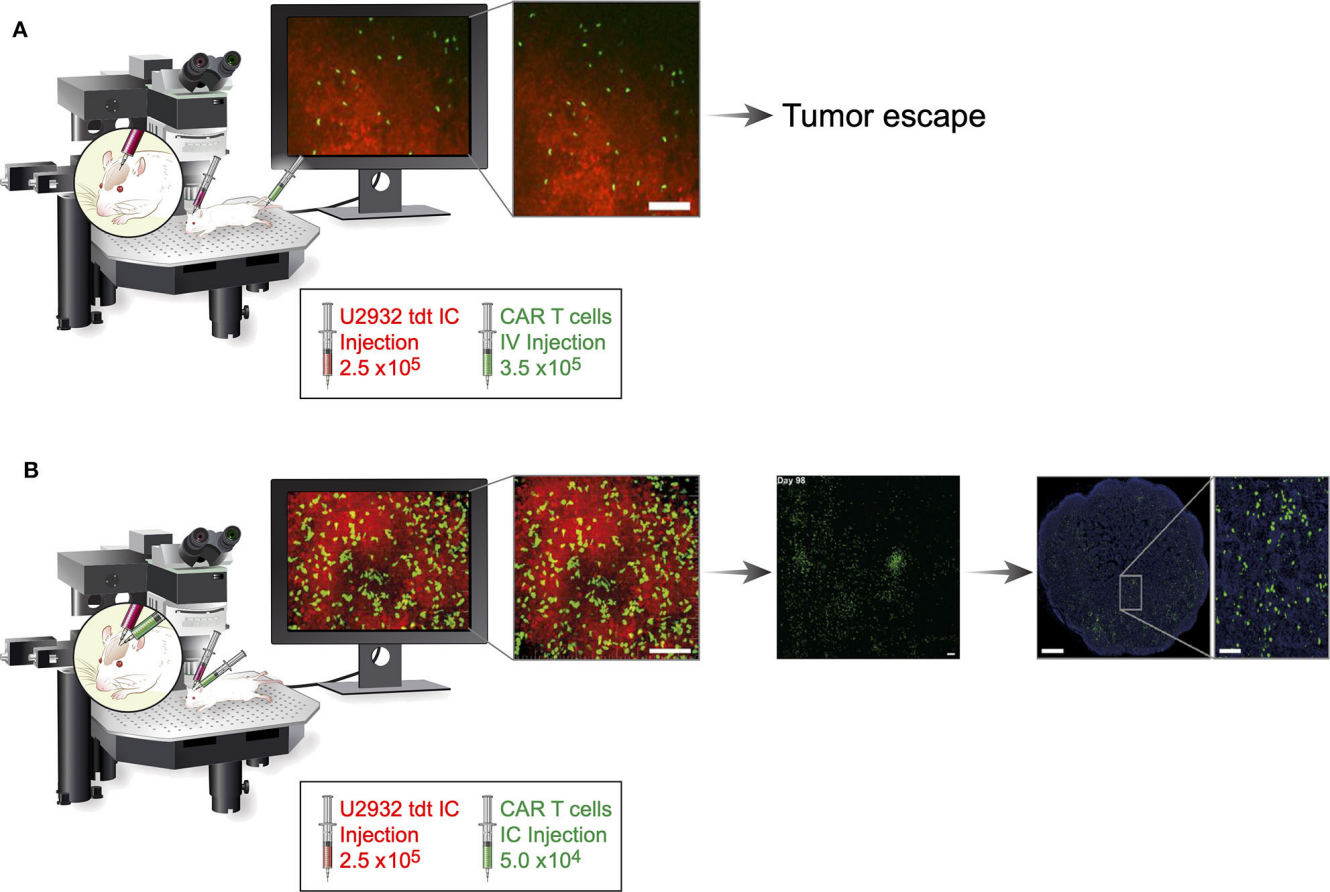
Schematic comparison of systemic and regionally delivered h19m28z CAR T cells in a PCNSL mouse model. Fourteen days after establishment of the cranial window, 2.5 ×10^6^ U2932 tdt (red) cells were injected (diluted from 1 μL of PBS to 2 μL of PBS) orthotopically in homozygous male NMRI Foxn1^nu/nu^ mice. Fourteen days after implementation of tumor cells, h19m28z CAR T cells (green) were administered either intravenously by injecting 3.5 ×10^5^ cells (diluted from 100 μL of PBS to 200 μL of PBS) or intracerebrally by injecting 5 ×10^4^ cells (diluted from 1 μL of PBS to 2 μL of PBS). **(A)** Intravenous administration resulted in a low presence (infiltration, accumulation, and depth) of h19m28z CAR T cells, without a sustained effect on tumor cells, in the majority of mice. **(B)** In contrast, after intracerebral injection, h19m28z CAR T cells were present at higher numbers and higher depth, compared with mock CAR T cells. h19m28z CAR T cells also had low velocity, compared with mock CAR T cells, owing to immune synapse formation and the killing of tumor cells, leading to reduced PCNSL growth and tumor regression. Moreover, compared with the mock CAR T cells, which persisted for <3 weeks, h19m28z CAR T cells were found to persist for up to 159 days (central panel) and migrated to non-draining lymph nodes (right panel). IC, intracerebral; IV, intravenous. This figure includes cropped figures from the article “Long-term in vivo microscopy of CAR T cell dynamics during eradication of CNS lymphoma in mice” by Mulazzani et al. (4), under Creative Commons CC-BY-NC-ND 4.0. Permission has been provided by authors of the original work to share modified material.

The most remarkable achievement of this study was its use of cranial windows and TPLS to observe intratumoral proliferation, CAR T-cell infiltration, and intracerebral CAR T-cell velocities at the tumor site without the need to sacrifice mice (Figure 1). It is likewise impressive that the study demonstrated that, although the non-activated CAR T-cell velocity in intracerebral tumors remained unchanged, antigen-activated anti-CD19 CAR T cells had reduced velocity, compared with mock CAR T cells. Reduced velocities suggest CAR T cells form an immunological synapse with tumor cells and that, after tumor eradication, intratumoral tumor-specific CAR T cells may resume higher velocities—observations that were associated with tumor regression. These observations were confirmed in mice without cranial windows, indicating that surgery-related inflammation did not influence results. The authors showed a difference in infiltration patterns. Whereas mock CAR T cells primarily accumulated at the tumor borders, h19m28z CAR T cells significantly outnumbered mock CAR T cells and infiltrated deeper tumor regions. Immunofluorescence analyses of harvested organs showed that anti-CD19 CAR T cells lacked CD27, a marker of differentiation from resting memory T cells to terminal effector T cells. In contrast, mock CAR T cells retained CD27. Even after tumor regression, intracerebral anti-CD19 CAR T cells were observed for 35 to 159 days. More importantly, following intracerebral injection, circulating anti-CD19 CAR T cells were observed in non-draining, distant inguinal lymph nodes at higher numbers than mock CAR T cells.

## Discussion

The study provides a comprehensive *in vivo* analysis of the mechanisms of CAR T cells in a PCNSL model, but the results raise questions that require further study. First, the authors showed that, in mice treated with mock CAR T cells, but not in mice treated with h19m28z CAR T cells, CD3 T cells were in close association with CD11c myeloid cells, which formed a dense rim around the tumor. The effects of tumoral stroma and the tumor immunosuppressive microenvironment on CAR T-cell migration, proliferation, and egress are not clear. These important factors that limit antitumor efficacy should therefore be studied in a solid-tumor model. The information obtained may not only highlight the influence of organ-specific immune microenvironments on CAR T cells but also allow investigators to identify strategies to manipulate the tumor immune microenvironment to further improve the antitumor efficacy of CAR T cells in solid tumors. Second, the authors show that the dose of CD19 CAR T cells delivered intracerebrally (2 ×10^4^ CAR T cells) was more effective than a 17.5-fold higher dose delivered intravenously (3.5 ×10^5^ CAR T cells). We and others have shown that, following regional administration of CAR T cells, CD4 CAR T cells are antigen-activated and can provide better helper function to CD8 CAR T cells (5); this results in higher antitumor efficacy, compared with systemic administration. Further dissection of the individual and interdependent roles of CD4 and CD8, as well as those of CAR and non-CAR T cells, within the solid-tumor environment following regional administration is therefore warranted. Third, it is not clear whether the entry of anti-CD19 CAR T cells into non-draining lymph nodes was influenced by cancer cells or whether the altered chemokine receptor profile of antigen-activated anti-CD19 CAR T cells facilitated their entry. A better understanding of CAR T-cell entry into tumors and lymph nodes will greatly influence solid-tumor CAR T-cell therapy. Fourth, understanding CAR T-cell dynamics in heterogenous antigen expression (6), which is common in solid tumors, unlike CD19 expression in hematological malignancies, is important. Fifth, we and others have shown that exhausted CAR T cells can be rescued by anti-PD1 strategies (6); as such, visualization of CAR T-cell kinetics following administration of anti-PD1 agent is relevant to current clinical applications (7).

The translational relevance of this study also raises questions for further investigation. Neurological toxicities, collectively referred to as CAR T cell-related encephalopathy syndrome, occur in approximately 12% to 32% of patients treated with CD19 CARs, highlighting the life-threatening risks of immune inflammatory reactions in the central nervous system (8, 9). Additionally, high-dose intravenous infusion (1 ×10^10^ T cells) increases the risk of serious pulmonary toxicities (10). Intracerebral delivery may reduce the risk of systemic toxicities and be more suitable for the treatment of PCNSL, a possibility that requires clinical investigation. Additionally, although understanding CAR T-cell dynamics within solid tumors and lymph nodes is important, it is highly desirable to correlate intratumoral and systemic CAR T-cell kinetics in a clinically relevant model, owing to limitations when obtaining serial biopsy specimens from patients.

The findings presented by the authors merit commendation for providing a framework for future investigation into the treatment of PCNSL as well as solid tumors.

## Author Contributions

HH, HQ, and PA wrote the draft. PA reviewed, corrected, and approved the final manuscript. All authors contributed to the article and approved the submitted version.

### Conflict of Interest

PA has received research funding from ATARA Biotherapeutics and OSE Immunotherapies, has received research fees from ATARA Biotherapeutics, and has an issued patent 10,538,588 (mesothelin-targeted chimeric antigen receptor) licensed to ATARA Biotherapeutics, issued patent EP1979000B1 (method for detection of cancer cells using virus), and pending patent applications WO2018165228A1, CA3034691A1, CA3007980A1, AU2016316033A1 (PD-1 dominant negative receptor), US20170172477A1 (wireless pulse-oximetry device), and on an *ex vivo* malignant pleural effusion culture system. The remaining authors declare that the research was conducted in the absence of any commercial or financial relationships that could be construed as a potential conflict of interest.
